# Complications following REM sleep behavior disorder

**DOI:** 10.1002/brb3.2129

**Published:** 2021-03-30

**Authors:** Eva Schaeffer, Daniela Berg

**Affiliations:** ^1^ Department of Neurology Christian‐Albrechts‐University of Kiel Kiel Germany; ^2^ Department of Neurodegeneration Hertie Institute for Clinical Brain Research University of Tübingen Tübingen Germany

**Keywords:** REM sleep behavior disorder, Parkinsons Disease

## Abstract

REM sleep behavior disorder (RBD) is gaining increasing attention as important prodromal marker for the development of neurodegenerative disorders such as Parkinson's Disease. However, the clinical relevance of this disorder and its association with other prodromal markers is often underestimated in clinical routine. We here report a case of severe clinical complications following extensive nocturnal movements due to RBD, aggravated by occurrence of additional prodromal non‐motor symptoms. This case stresses the importance of recognizing RBD as a multisystem disease by all clinical disciplines.

## Graphical abstract

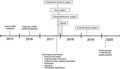

## 1 INTRODUCTION

Idiopathic REM sleep behavior disorder (iRBD) has increasingly gained importance as the most relevant prodromal marker for alpha‐synucleinopathies, with more than 80% of the affected developing Parkinson's Disease (PD), Dementia with Lewy bodies or Multiple System Atrophy later in life (Postuma et al., 2019). The uncontrolled nocturnal movements occurring in RBD can lead to severe injuries. However, although specialists in Neurology and Sleep Medicine are meanwhile aware of RBD and its implications, there is still a lack of knowledge in other medical disciplines and the relevance of this parasomnia is often underestimated. Moreover, although the association of RBD with other prodromal factors such as constipation is well known (Barber et al., 2017), current treatment guidelines do not take these co‐factors into account.

We here describe a patient who suffered from severe complications of nocturnal behavior due to RBD, which became life‐threatening by associated prodromal symptoms.

## 2 CASE DESCRIPTION

The male patient was diagnosed with RBD (confirmed by polysomnography) when he was 63 years. At first presentation at our Department at the age of 64, he showed no cardinal motor symptoms. However, history and neurological examination revealed several other prodromal markers for PD, including hyposmia, orthostatic hypotension, depression, subjective mild cognitive impairment, and irregularities in bowel movements. The patient had decided for a treatment with melatonin (4mg) and amitriptyline (75mg/d), which was started after the first onset of RBD to improve depressive symptoms. The explicit advice of caution regarding a potential aggravation of RBD symptoms by antidepressants was rejected by the patient as he had the impression of benefitting without any side effects.

One month later, the patient fractured his femoral neck acting out a dream, resulting in immediate surgery. Four days later, he developed signs of a severe ileus, followed by a second emergency surgery. Postoperatively, the patient suffered from wound dehiscence, leading to a third surgery. Finally, the patient developed a severe delirium, which necessitated a prolonged stay in geriatrics. After two months of hospitalization and another month in a rehabilitation clinic, the patient could finally be discharged.

**FIGURE 1:**
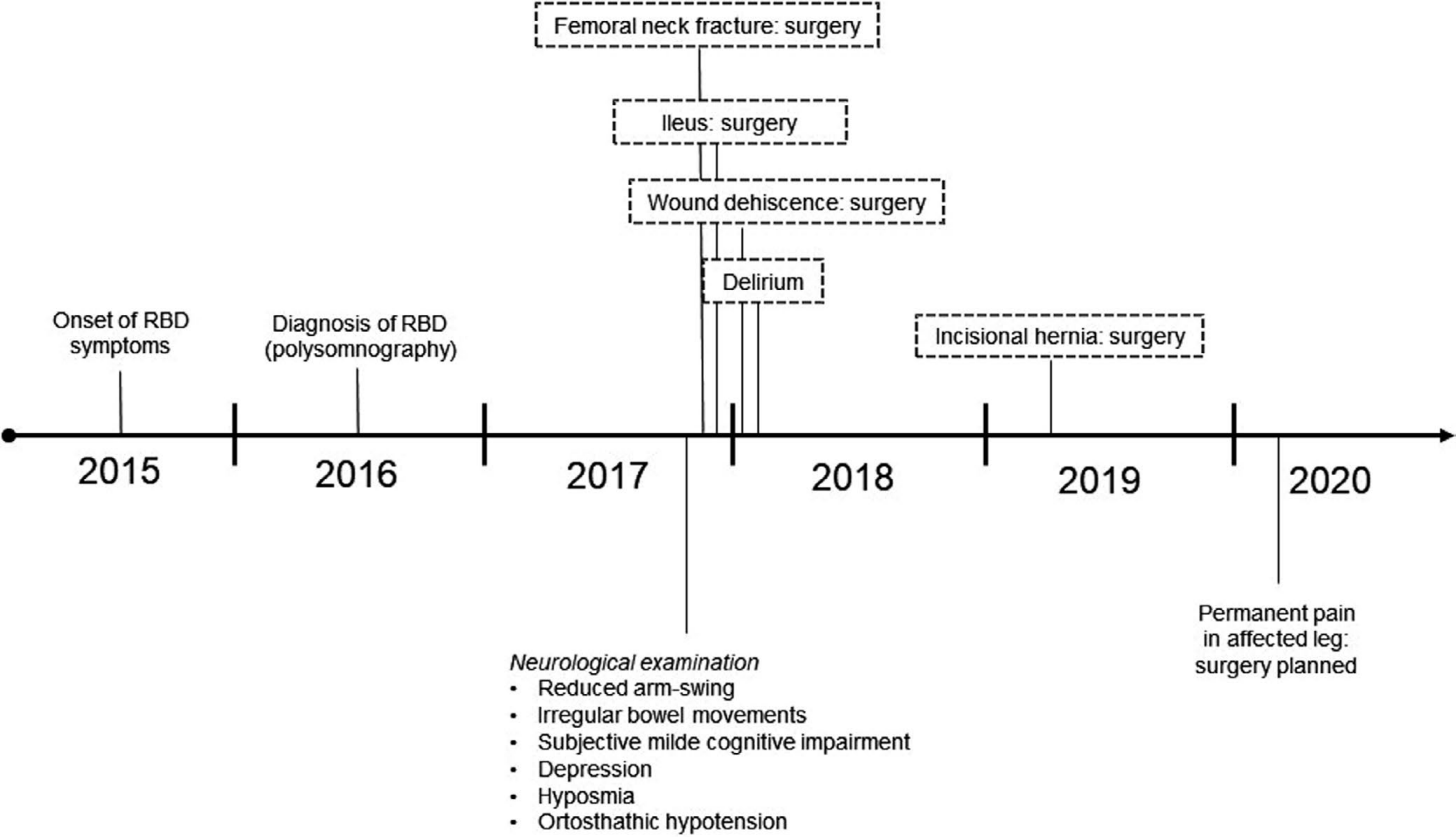
Timeline of disease course

Clinical follow‐ups revealed a secondary abdominal hernia leading to a fourth surgery one year later. The patient is to date still suffering from pain in the primarily leg and is considering a fifth surgery (Figure 1).

## 3 DISCUSSION

This case demonstrates the importance of taking additional symptoms associated with probable prodromal PD seriously. Clinical and pathological studies have shown that the occurrence of RBD may constitute a specific subtype of PD, with a proposed start of pathology in the enteral nervous system followed by widespread neurodegeneration, leading to a higher risk of cognitive decline and a higher risk for malign clinical progression (Fereshtehnejad et al., 2015), leading to the assumption that in these patients multiple parts of the nervous system are already affected in the prodromal phase of PD.

In this case, the rapid development of a severe ileus during immobility can be seen as result of the pre‐existing constipation—a frequent prodromal symptom in RBD patients. Moreover, considering the higher rates of a postoperative delirium in individuals with clinical PD, it can be assumed that a condition of prodromal PD might also influence this risk.

We conclude that it is of high relevance to pay attention to potentially associated other prodromal symptoms in RBD patients. In the present case, none of the medical reports from anesthesia, surgery, geriatrics, and rehabilitation mentioned the presence of RBD in our patient, although it was clearly the cause of all following medical conditions. It therefore is an important need to strengthen awareness for RBD and potential associated symptoms in all medical disciplines.

## CONFLICTS OF INTEREST

E. Schaeffer received intramural research funding from the University of Kiel and speaker's honoraria from Bayer Vital GmbH and Novartis, outside the submitted work. D. Berg reports grants from Janssen Pharmaceutica, grants from Damp foundation, grants from German Parkinson's Disease Association (dPV), grants from BMWi, grants from BMBF, grants from Parkinson Fonds Deutschland GmbH, grants and speaker's honoraria from and consultancies for UCB Pharma GmbH, grants and speaker's honoraria from TEVA Pharma GmbH, grants from and consultancies for Novartis Pharma GmbH, grants and speaker's honoraria from and consultancies for Lundbeck, speaker's honoraria from and consultancies for BIAL, speaker's honoraria from and consultancies for Biogen, honoraria from Bayer and Zambon, outside the submitted work.

### PEER REVIEW

The peer review history for this article is available at https://publons.com/publon/10.1002/brb3.2129.

## References

[brb32129-bib-0001] Barber, T. R. , Lawton, M. , Rolinski, M. , Evetts, S. , Baig, F. , Ruffmann, C. , Gornall, A. , Klein, J. C. , Lo, C. , Dennis, G. , Bandmann, O. , Quinnell, T. , Zaiwalla, Z. , Ben‐Shlomo, Y. , & Hu, M. T. M. (2017). Prodromal Parkinsonism and neurodegenerative risk stratification in REM sleep behavior disorder. Sleep, 40(8), zsx071. 10.1093/sleep/zsx071. PMC5806544 28472425

[brb32129-bib-0002] Fereshtehnejad, S.‐M. , Romenets, S. R. , Anang, J. B. M. , Latreille, V. , Gagnon, J.‐F. , & Postuma, R. B. (2015). New clinical subtypes of Parkinson disease and their longitudinal progression: A prospective cohort comparison with other phenotypes. JAMA Neurol., 72(8), 863–873. 10.1001/jamaneurol.2015.0703. 26076039

[brb32129-bib-0003] Postuma, R. B. , Iranzo, A. , Hu, M. , Högl, B. , Boeve, B. F. , Manni, R. , Oertel, W. H. , Arnulf, I. , Ferini‐Strambi, L. , Puligheddu, M. , Antelmi, E. , Cochen De Cock, V. , Arnaldi, D. , Mollenhauer, B. , Videnovic, A. , Sonka, K. , Jung, K.‐Y. , Kunz, D. , Dauvilliers, Y. , … Pelletier, A. (2019). Risk and predictors of dementia and parkinsonism in idiopathic REM sleep behaviour disorder: A multicentre study. Brain, 142(3), 744–759. 10.1093/brain/awz030. 30789229 PMC6391615

